# *kin-3* genetically suppresses *sur-6* in centrosome assembly during *Caenorhabditis elegans* embryogenesis

**DOI:** 10.17912/micropub.biology.000791

**Published:** 2023-03-07

**Authors:** Jeffrey C. Medley, Mi Hye Song

**Affiliations:** 1 Department of Biological Sciences, Oakland University, Rochester, Michigan, United States

## Abstract

Protein phosphorylation plays a critical role in cell cycle progression. In *Caenorhabditis elegans* , Casein Kinase II (CK2) negatively regulates centrosome assembly, and Protein Phosphatase 2A (PP2A) ^SUR-6 /B55^ acts as a positive regulator of centrosome duplication, suggesting CK2 and PP2A ^SUR-6 /B55^ play opposing roles in centrosome assembly. Here, we examined the genetic interaction between *kin-3 ,* encoding the catalytic subunit of CK2, and *sur-6 ,* encoding the PP2Aregulatory subunit SUR-6 /B55, in *Caenorhabditis elegans* embryos. Our results show that *kin-3 (RNAi)* partially restores normal centrosome duplication and embryonic viability to hypomorphic *sur-6* mutants, suggesting that *kin-3* genetically suppresses *sur-6* in centrosome assembly during *Caenorhabditis elegans* embryogenesis.

**Figure 1. kin-3(RNAi) suppresses embryonic lethality and abnormal centrosome behavior in sur-6(or550) mutant embryos:**
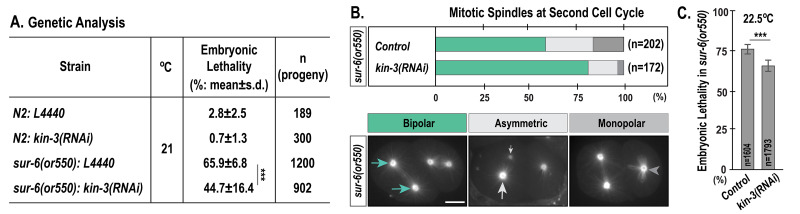
Genetic analysis of *sur-6(or550)* mutants at the semi-restrictive temperature (21°C, *** *p* <0.001) **(B)** Quantification of mitotic spindle formation (stacked bars) during the second cell cycle in temperature-sensitive *sur-6(or550)* mutants. At 22.5°C, *sur-6(or550)* embryos often display defective centrosome duplication, with monopolar (dark gray) or asymmetric bipolar (light gray) spindles during the second mitosis. *kin-3(RNAi)* increases the percentage of symmetric bipolar spindles (green) in *sur-6(or550)* embryos. n is the number of blastomeres scored. The bottom panel illustrates representative embryos expressing mCherry::tubulin, with symmetric bipolar (green arrows), asymmetric bipolar (light gray arrows), and monopolar spindles (dark gray arrowheads) at the second mitosis in *sur-6(or550)* mutants, indicative of defective centrosome assembly during the first cell cycle. **(C) %** Embryonic lethality of *sur-6(or550)* mutant animals at 22.5°C. Data are presented as mean±s.d. n is the number of progeny scored. (*** *p* <0.0001)

## Description

Protein Phosphatase 2A (PP2A) has been shown to play a conserved role in positively regulating centrosome assembly in different systems, including *Caenorhabditis elegans,* flies and human cells (Brownlee et al., 2011; Kitagawa et al., 2011; Song et al., 2011). PP2A functions as a trimeric holoenzyme composed of a catalytic subunit, a scaffolding subunit, and one of the various regulatory subunits (Fowle et al., 2019). *C. elegans* SUR-6 , the homolog of the human PP2A B55 regulatory subunit, has been shown to direct the role of PP2A in centrosome assembly (Kitagawa et al., 2011; Song et al., 2011). On the other hand, Casein Kinase II (CK2) has been reported to negatively regulate centrosome duplication in *C. elegans* embryos (Medley et al., 2017). CK2 is an evolutionarily conserved serine/threonine protein kinase that functions as a tetrameric holoenzyme comprising two catalytic (CK2α: KIN-3 in *C. elegans* ) and two regulatory (CK2β: KIN-10 in *C. elegans* ) subunits (Hu and Rubin, 1990; Hu and Rubin, 1991; Niefind et al., 2009). The previous findings in *C. elegans* suggest that CK2 and PP2A play an opposing role in centrosome assembly during *C. elegans* embryonic development.

To examine the functional interaction between CK2/ KIN-3 and PP2A ^SUR-6 /B55^ in *C. elegans* embryos, we first asked how *kin-3 (RNAi)* -mediated inhibition of CK2 activity might affect the embryonic lethal phenotype in the hypomorphic *sur-6 ( or550 )* mutant. The *sur-6 ( or550 )* allele is a recessive temperature-sensitive mutation that produces a highly penetrant embryonic lethal phenotype at the permissive (27% at 15°C) and restrictive (84% at 26°C) temperature conditions (O’Rourke et al., 2011). Consistent with previous studies (Alessi et al., 2015; Medley et al., 2017), we observed partial depletion of CK2/ KIN-3 by *kin-3 (RNAi)* feeding for 24 hours did not produce embryonic lethality in wildtype ( N2 ) animals (0.7 ± 1.3%) grown at 21°C, the semi-restrictive temperature condition for the *sur-6 ( or550 )* allele ( **Fig. 1A** ). However, under the same condition, *kin-3 (RNAi)* significantly reduced the embryonic lethality of *sur-6 ( or550 )* mutants (44.7 ± 16.4%, *p* <0.001), compared to control RNAi-treated *sur-6 ( or550 )* mutants (65.9 ± 6.8%) ( **Fig. 1A** ). These results show that *kin-3 (RNAi)* suppresses the embryonic lethal phenotype of the *sur-6 ( or550 )* mutant allele *.*

Next, we examined how *kin-3 (RNAi)* affected centrosome assembly in the *sur-6 ( or550 )* mutant embryo ( **Fig. 1B** ). SUR-6 /B55, as part of PP2A holoenzyme, has been shown to function as a positive regulator of centrosome duplication during *C. elegans* embryonic cell divisions (Kitagawa et al., 2011; Song et al., 2011). The *sur-6 ( or550 )* mutation results in significant embryonic lethality, and the *sur-6 ( or550 )* allele fails to complement *sur-6 ( sv30 )* that exhibits abnormal centrosome behavior (Kitagawa et al., 2011; O’Rourke et al., 2011). In addition, it has been shown that *sur-6 (RNAi)-* treated wildtype embryos exhibit a high rate of asymmetric bipolar spindles and that *sur-6 (RNAi)* -treated *zyg-1 ( it25 )* embryos grown at the permis­sive temperature display a significant rate of monopolar spindles (Table S1 in Song et al., 2011). However, at the permissive temperature, *zyg-1 ( it25 )* mutant embryos exhibit 100% bipolar spindles (O’Connell et al. 2001). In this study, we observed that *sur-6 ( or550 )* mutant embryos grown at the semi-restrictive temperature (22.5°C) exhibit monopolar spindles and asymmetric bipolar spindles during the second mitosis, likely resulting from defective centrosome assembly during the first mitotic division ( **Fig 1B** ). At 22.5°C, control RNAi-treated *sur-6 ( or550 )* embryos produced 58.5 % symmetric bipolar, 25.3% asymmetric bipolar, and 16.2% monopolar spindles at the second mitosis (n=202 blastomeres), whereas *kin-3 (RNAi)* -treated *sur-6 ( or550 )* embryos displayed 81% symmetric bipolar, 16% asymmetric bipolar, and 3% monopolar spindles (n=172 blastomeres), suggesting that *kin-3 (RNAi)* partially restores normal centrosome assembly to *sur-6 ( or550 )* embryos ( **Fig 1B** ). Consistent with the effect of *kin-3 (RNAi)* -mediated knockdown in centrosome duplication, *kin-3 (RNAi)* led to a significant decrease in the embryonic lethality of *sur-6 ( or550 )* mutants (66.4 ± 7 %, n=1793; *p* <0001), compared to control RNAi-treated *sur-6 ( or550 )* animals (76.0 ± 6%, n=1604) grown at 22.5°C ( **Fig 1C** ).

Together, our results illustrate that *kin-3 (RNAi)-* mediated inhibition of KIN-3 partially restores normal centrosome duplication and successful embryogenesis to *sur-6 ( or550 )* mutants. CK2 may directly antagonize PP2A ^SUR-6 /B55^ activity to regulate centrosome assembly. Another possibility is that CK2 and PP2A ^SUR-6 /B55^ counteract to regulate the phosphorylation state of a centrosome factor. PP2A ^SUR-6 /B55^ and CK2 have been shown to regulate ZYG-1 , a key centrosome factor (Medley et al., 2017; O’Connell et al., 2001; Song et al., 2011). Thus, it is intriguing to speculate that PP2A ^SUR-6 /B55^ and CK2 regulate centrosome duplication by targeting ZYG-1 . However, additional studies will be required to fully understand how CK2 and PP2A ^SUR-6 /B55^ coordinate to regulate centrosome assembly.

## Methods

***C. elegans* Culture and RNAi feeding**

All strains were derived from the wildtype N2 strain and maintained at 20°C on MYOB plates seeded with *Escherichia coil* OP50 . The L4440 empty vector was used as a negative control *,* and RNAi feeding was performed as previously described (Kamath et al., 2003; Medley et al., 2017). For embryonic viability assays by RNAi feeding, L4 animals were singled to individual plates and allowed to self-fertilize for 24 hours at the indicated temperature condition. All progeny were allowed at least 24 hours to complete embryogenesis before scoring the number of hatched larvae and unhatched (dead) eggs. For centrosome duplication events, L4 animals were grown in control RNAi or *kin-3 (RNAi)* plates for 16-18 hours at 22.5°C, adult gravid worms were mounted, and embryos were scored for mitotic spindle formation.

**Cytological Analysis**

Confocal microscopy was performed as described previously (Medley et al., 2017) using a Nikon Eclipse Ti-U microscope equipped with a Plan Apo 60×1.4 NA lens, a spinning disk confocal (CSU X1), and a Photometrics Evolve 512 camera. For live imaging, we used 3% (w/v) agarose in Egg Buffer to mount embryos. MetaMorph software was used for image acquisition, and Adobe Photoshop/Illustrator 2023 for image processing.

**Statistical Analysis**

All *p* -values were calculated using two-tailed t-tests assuming equal variance among sample groups. Statistics were produced using R statistical software and presented as mean ± standard deviation (s.d.).

## Reagents

**Table:** 

Strain	Genotype	Available
N2	wildtype	CGC
EU1062	*sur-6 ( or550 ) *	CGC
MTU849	*sur-6 ( or550 ); bsIs15 [pNP99:unc-119(+) tbb-1p::mCherry::tbb-2::tbb-2 3’-utr] *	This study
